# Effects of the combination of the gratitude extension-construction theory and peer interactive education on self-management ability and quality of life in school-age children with asthma in China

**DOI:** 10.3389/fped.2025.1678459

**Published:** 2025-11-17

**Authors:** Lijin Gao, Lijia Liu, Wen Zhang, Haixin Chen, Xiuming Wei, Yawei Guo, Xiaohua Zhou, Yibo Zheng

**Affiliations:** Department of Pediatrics, Bethune International Peace Hospital, Shijiazhuang, Hebei, China

**Keywords:** asthma, school-age, children, gratitude extension-construction theory, peer interactive education, self-management

## Abstract

**Objective:**

To explore the effects of the combination of the gratitude extension-construction theory and peer interactive education on self-management ability and quality of life in school-age children with asthma in China.

**Methods:**

A total of 120 children with bronchial asthma admitted to Bethune International Peace Hospital, Shijiazhuang, China from January 2023 to March 2024 were selected and randomly divided into study group and control group. The control group accepted routine treatment and nursing. Based on routine treatment and nursing, the study group accepted the combination of the gratitude extension-construction theory and peer interactive education. The self-management ability, asthma control score, lung function, quality of life, recurrence, nursing compliance and nursing satisfaction were compared in both groups.

**Results:**

Compared to before intervention, the self-management ability scores, C-ACT score, levels of lung function indexes and PAQLQ scores were elevated at 6 and 12 months after intervention (*P* < 0.05). Compared to the control group, the study group had higher self-management ability scores, C-ACT score, levels of lung function indexes and PAQLQ scores at 6 and 12 months after intervention (*P* < 0.05). Compared to the control group, the study group had lower frequency of asthma attacks, emergency department and hospitalization within 12 months after treatment, better nursing compliance and higher nursing satisfaction (*P* < 0.05).

**Conclusion:**

The combination of the gratitude extension-construction theory and peer interactive education significantly promote the self-management ability, improve lung function, promote the quality of life, reduce the recurrence, and enhance the nursing compliance and nursing satisfaction of school-age children with asthma in China.

## 1 Introduction

Bronchial asthma is a common chronic airway inflammatory disease in clinical practice (1). Epidemiological studies have shown that school-age children have a high incidence of bronchial asthma, with the prevalence rate ranging from 0.12% to 3.41%, and the trend is increasing (2). At present, the clinical treatment of bronchial asthma lacks specific drugs and treatment methods, and cannot solve the pathogenic factors of asthma from the root (3). At the same time, the symptom control of children with asthma in China is still not ideal, at least 20% of children with asthma symptoms cannot be well controlled (4). Poor symptom control of school-age children with asthma will not only increase the probability of medical treatment and hospitalization, but also seriously affect their academic and mental health, and increase the family's economic and social medical burden (5).

Inadequate understanding of the disease and treatment of children with asthma and their guardians, as well as the resulting non-standard treatment and poor compliance, are important reasons affecting the level of asthma control in children (6). Therefore, it is crucial to improve the self-management ability and compliance of children and families through asthma education. Foreign studies have also confirmed the correlation between asthma education and treatment compliance and disease control level (7). In addition to improving physical health and effectively reducing the consumption of emergency medical treatment and basic medical resources, effective asthma education can also reduce the occurrence of disease-related psychological adverse events and social disorders of patients and their families (8).

As children grow older, their ability to observe, learn and want to learn increases, especially in school-age children, and children with asthma pay more attention to their own health problems (9). The survey shows that school-age children have a certain amount of health information, but the mastery of disease-related knowledge is generally not high (10). They are eager to master disease knowledge such as the treatment of acute attacks and self-monitoring methods, so school-age children have great potential in the subjective initiative of self-management of chronic diseases (11). Foreign studies have pointed out that interactive asthma education based on school environment can significantly and permanently enrich the asthma knowledge of school-age children, improve children's asthma self-management ability, and improve the level of disease control (12).

As a positive psychology theory, gratitude extension-construction theory emphasizes that positive emotions are mainly derived from evolution and have obvious purpose and adaptability (13). This theory states that gratitude is a positive emotion that increases happiness and satisfaction by focusing on the positive aspects of one's life, such as giving, helping, and enjoying (14). It has been that the nursing program based on the gratitude extension-construction theory can improve the gratitude level of cervical cancer patients, alleviate negative emotions, and improve their quality of life (15). However, the effect of nursing under the gratitude extension-construction theory on self-management ability and quality of life in school-age children with asthma remains unclear.

Therefore, this study combined gratitude extension-construction theory with peer interactive education for disease management of school-age children with asthma, and evaluated the impact of management on the treatment effect, self-care ability, treatment compliance and hospitalization frequency of children with asthma, providing evidence for the management of school-age children with asthma in China.

## 2 Data and methods

### 2.1 General data

A total of 120 children with bronchial asthma admitted to Bethune International Peace Hospital, Shijiazhuang, China from January 2023 to March 2024 were selected and randomly divided into study group and control group, with 60 children in each group. Inclusion criteria: (1) Children met the diagnostic criteria of the Guidelines for the Diagnosis and Prevention of Bronchial Asthma in Children; (2) Age 6–14 years old; (3) All children were treated with the treatment regimen recommended by the Global Initiative for Asthma Control (GINA); (4) Children had certain cognitive ability and communication ability. Exclusion criteria: (1) Children in the acute attack period; (2) Combined with serious infectious diseases and internal diseases; (3) Combined with other respiratory diseases; (4) Combined with mental illness; (5) Children had defects in language expression; (6) Children who lost follow-up or dropped out. The parents of children were informed of the details of the study and agreed to participate.

### 2.2 Randomization

Random grouping was carried out using a computer-generated random number table. To ensure the scientificity and fairness of the random grouping process, a specialized staff was appointed to handle this operation. The specific operation steps were as follows: The 120 children with bronchial asthma who met the inclusion criteria were numbered sequentially from 1 to 120 according to the order of enrollment. Using the random number generation function in professional statistical software (such as SPSS 25.0), 120 random numbers within the range of 0–1 were generated. The generated random numbers were matched with the patient numbers and sorted in ascending order. Then, the sorted patients were divided into the study group and the control group in equal proportions, with 60 patients in each group. The grouping results were sealed in an opaque envelope and kept by a designated person.

### 2.3 Blinding

When recruiting the children and their parents, the specific information about the group allocation was not disclosed to them. They were only informed that they would participate in a research on educational intervention for children with asthma. The study group and the control group would receive different educational methods, but the specific advantages of each method or the detailed differences were not mentioned to avoid the participants developing expectation biases due to knowing the group allocation, which might affect their behaviors and self-reported results.

During the research process, the parents were also unaware of the specific groupings of the children. Throughout the study, the parents were only required to provide routine care for the children and cooperate with the researchers to complete some necessary information collection tasks, such as recording the children's daily symptoms and medication usage. However, they were not informed of the relationship between these information and the groupings, to prevent the parents' subjective attitudes and behaviors from having an indirect impact on the children.

The result evaluators do not participate in the grouping process of the children and the implementation process of the intervention. Before the assessment, a dedicated person will sort out the assessment materials of the children, remove the identifiers that may reveal the grouping information, and only retain the basic information and assessment data required for the assessment. The evaluators assess the children's self-management ability and quality of life according to the unified standards and procedures, without knowing whether the children belong to the study group or the control group, in order to ensure the objectivity and fairness of the assessment results.

## 3 Management methods

The control group received routine treatment and nursing, and the nurses helped the children and their parents understand their condition and treatment plan, and answered questions.

Based on routine treatment and nursing, the study group implemented the management method combining the gratitude extension-construction theory and peer interactive education.

The implementation of the gratitude extension-construction theory was as follows:

### 3.1 Member training

The team consisted of 1 head nurse of pediatrics and 5 responsible nurses of pediatrics. The pediatric head nurse has 10 years of experience in pediatric nursing management, possessing rich clinical nursing knowledge and team leadership skills. She has participated in the organization and teaching of internal hospital nursing training on multiple occasions and has extensive experience in the field of nursing training. The 5 pediatric responsibility nurses all have 5 years or more of clinical pediatric nursing experience. Among them, 3 nurses have a bachelor's degree and 2 nurses have a junior college degree. They have accumulated a lot of experience in communicating with children and their parents in daily nursing work, are familiar with the nursing procedures for common pediatric diseases, and can skillfully handle various emergencies. They have accumulated a great deal of experience in communicating with patients and their parents during daily care. They are familiar with the nursing procedures for common pediatric diseases and are capable of handling various emergencies proficiently.

All members participated in the training of gratitude extension-construction theory and practical operation skills. The training procedure was as follows:

A training instructor team was established, consisting of 1 external expert with rich theoretical knowledge and practical experience in gratitude expansion construction, and the head nurse of the pediatric care team from our research team. These two individuals jointly served as the training instructors. Based on the gratitude expansion construction theory and the actual operation requirements of pediatric care, a detailed training plan was formulated, clearly defining the training objectives, contents, time schedule, and assessment methods. The training content covered the basic knowledge of gratitude expansion construction theory, application cases in pediatric care, and key points of practical operation skills. The teaching materials included theoretical handouts, operation manuals, and related video materials; teaching aids included models and props; the simulation scene equipment was set up according to common pediatric care scenarios, such as wards and treatment rooms.

The theoretical training was conducted through centralized lectures. External experts and head nurses take turned to explain the basic knowledge of the gratitude expansion construction theory, as well as its application principles and methods in pediatric nursing. During the lectures, actual cases were used for analysis and discussion, guiding the participants to think actively and ask questions, ensuring that they understand the theoretical knowledge. The theoretical training is scheduled for 4 class hours and was conducted over 28 days.

The practical operation skills training was conducted in simulated scenarios, with the head nurse and external experts providing on-site demonstrations to build the relevant nursing operation skills, such as communication skills with patients and their parents, and ways to guide patients to express gratitude. After the demonstration, the members were divided into groups to practice the actual operations, with the instructor providing guidance and promptly correcting the members' operational errors to ensure they master the correct operation methods. The practical operation skills training was scheduled for 4 class hours and is carried out over 28 days.

Before the training began, an initial test on theoretical knowledge and practical operational skills was conducted for all members to understand their basic levels, so that targeted training could be carried out subsequently. The theoretical assessment was conducted through written tests, with question types including multiple-choice questions, fill-in-the-blank questions and short-answer questions; the practical operation assessment was carried out in simulated scenarios, and the scores were evaluated by the instructors based on the pre-defined scoring criteria. During the training process, the members' progress and mastery were evaluated through methods such as classroom questioning, group discussions, and practical operation exercises. This enabled us to promptly understand the members' learning progress and mastery level, and adjust the training schedule and methods accordingly. After the training, a theoretical and practical operation assessment was conducted for all members. The theoretical re-assessment was carried out one week after the training and the results of this assessment was compared with the initial test results to analyze the members' mastery and consolidation of knowledge. If the members' scores significantly decreased or they failed to accurately understand the key points, targeted one-on-one tutoring would be arranged. The practical operation simulation assessment set up a simulated scenario and required team members to perform actual operations based on the knowledge learned during the training. An assessment team consisting of the head nurse and external experts scored the operation according to the pre-established surgical scoring criteria. The assessment content included the standardization of the surgical process, the effectiveness of communication with patients and parents, and the ability to handle emergencies. Any deficiencies identified during the assessment would receive timely feedback and guidance, and the relevant personnel needed to undergo further simulation practice until they reached the qualified standard.

To maintain consistency in data collection and patient care, we implemented the following measures: (1) Developing detailed nursing work procedures and operational guidelines, clearly specifying the specific steps and requirements for each nursing process. (2) Establishing a standard process for data collection, clearly defining the time, location, method and content of data collection. (3) Developing a checklist for nursing operations, listing the key points and precautions for each nursing step. During the nursing operations, nurses checked against the checklist to ensure that each step was executed correctly. (4) Designing a data collection checklist to monitor and inspect the process of data collection. The checklist included whether the data collection was complete and accurate, and whether the records were clear. The nurses regularly reviewed the data collection checklist and promptly identified and corrected any problems that arose during the data collection process. (5) The training sessions were regularly organized for members on standardized procedures and checklists to ensure they were familiar with and able to master the relevant content. The training methods included group lectures, case studies, and simulation exercises. Through these training sessions, the members' operational skills and data collection abilities were continuously improved. (6) The head nurse and external experts regularly supervised and inspected the members' nursing work and data collection processes. Any identified problems were promptly reported and guided. At the same time, a feedback mechanism was established to encourage mutual supervision and communication among the members, aiming to jointly improve the quality of nursing and the accuracy of data collection.

All members completed the study and passed the assessment. To ensure the quality of the training and the proper implementation of the subsequent intervention measures, after the training was completed, the following monitoring was conducted for the participants: (A) Re-examination of theoretical assessment: One week after the training concludes, a theoretical assessment was conducted again for the members. The results of this assessment were compared with those of the initial test to analyze the members' mastery and consolidation of knowledge. If there was a significant decline in the members' scores or if they did not accurately understand key points, targeted one-on-one tutoring was arranged. (B) Actual operation simulation assessment: A simulation scenario was set up, requiring team members to perform actual operations based on what they had learned during the training. An assessment team consisting of the head nurse and external experts scored the operations according to the pre-established operation scoring criteria. The assessment content included the standardization of the operation process, the effectiveness of communication with patients and parents, and the ability to handle emergencies. For any deficiencies identified during the assessment, timely feedback and guidance were provided, and members were required to conduct further simulation practice until they met the qualified standards.

### 3.2 Construction and intervention

The intervention plan was developed according to the situation of the department and modified continuously. After discussion, the plan was carried out.

#### 3.2.1 In hospital

(A) Motivational talk (symposium), on the day of admission or the second day, the nurses organized the children and their parents to attend a symposium, watch the recorded video of the family experience of asthma, invite 1–2 groups of children with better disease control and their parents to share their experience, and the responsible nurse guided the newly admitted children and their parents to establish positive emotions.
  To monitor the compliance of nurses with the intervention protocol and the adherence of the children and their parents, we set up recording and video equipment at the seminar site to fully document the entire process. A dedicated person conducted regular audits of the recorded videos and audio, checking whether the nurses organized the seminar in accordance with the planned procedures, including whether the recorded videos were played accurately, whether the invited sharing families met the requirements, and whether they effectively guided the newly admitted children and their parents to build positive emotions. At the same time, we counted the number of participants in each seminar, the participation levels of the children and parents (such as questions asked, interaction situations), to evaluate the implementation effect of the seminar. In addition, the responsible nurse was required to fill out the self-inspection form after each seminar, recording the implementation details of the seminar, the problems encountered and the solutions. At the same time, nurses were encouraged to conduct mutual inspections among themselves, exchange experiences and propose improvement suggestions. In addition, the head nurse randomly selected 30% of the seminar videos and the corresponding self-checklists every month. Using a consistency checklist, a detailed review was conducted to verify whether the content recorded by the nurses was consistent with the actual presentation in the videos. Any inconsistencies found were promptly communicated with the responsible nurses and corrective actions were required.
(B) Positive reminiscence and hope (bedside talk), once a day, 10 min/time, the responsible nurse guided the children and their parents to recall the good experience in the past, look forward to the positive experience in the future, and think about the efforts that need to be made for it.
  After each bedside communication session, the responsible nurse immediately recorded the content of the communication, the child's reaction, the parent's reaction, the problems encountered during the guidance process, and the solutions adopted on a dedicated record sheet. The head nurse reviewed these record sheets every week, using a consistency checklist to check whether the nurses had conducted the communication as agreed (such as whether the communication duration reached 10 min) and whether the content of the communication met the standards (whether it focused on positive memories and hopes). For incomplete or non-compliant records, timely communication was conducted with the responsible nurse and rectification was required. The head nurse randomly selected 20% of the children from time to time and had conversations with the children and their parents to understand the implementation of bedside talks. This included checking if the frequency of the talks was once a day, whether the nurses' guidance methods were appropriate (judging by asking the parents and children about their feelings towards the nurses' guidance methods), and the feelings of the children and their parents, in order to obtain more accurate information.
(C) Gratitude meditation and expression (bedside talk), once a day, 10 min/time, the responsible nurse guided the children and their parents to think about happy moments, things and people in life, and expressed gratitude to their relatives and friends through words, videos, cards and other means.
  After each communication session, the nurses filled out a dedicated record sheet to document the details of the interaction. The head nurse reviewed the record sheets weekly and used a consistency checklist to verify whether the duration and content of the communication were centered around the themes of gratitude meditation and expression. Any records that did not meet the requirements were promptly communicated and rectified. The head nurse randomly selected 20% of the children from time to time and conducts conversations with the children and their parents to understand the frequency of the conversations, whether the nurse's guidance methods were appropriate, and the feelings of the children and their parents.
(D) Life essay (bedside talk), once a day, 10 min/time, the responsible nurse guided the children and the parents to record the beauty of life through diaries, drawings, essays and other forms, express their expectations for the future, and communicate and record together.
  After each bedside communication session, the responsible nurse recorded the content of the communication, the child's reaction, the parents' reaction, the problems encountered during the guidance process, and the solutions in a dedicated record sheet. The head nurse reviewed the record sheets every week, using a consistency checklist to check whether the duration and content of the communication meet the requirements (whether they revolved around the theme of life notes), and promptly communicated and corrected any incomplete or non-compliant records. The head nurse randomly selected 20% of the children from time to time and conducted conversations with the children and their parents to understand the frequency of the conversations, whether the nurse's guidance methods were appropriate, and the feelings of the children and their parents.
(E) Interest support and encouragement (in any form), the responsible nurse encouraged the children and their parents to find a comfortable environment in the hospital at any time to develop their own interests, such as reading, listening to music, playing games, and painting, and prepared toys and books suitable for the children in the ward to help them divert their attention.
  We conducted regular inspections of the wards to ensure that there were sufficient toys and books suitable for the children, that the environment of the wards was comfortable and safe, and that it was conducive to the children's development of hobbies. At the same time, we recorded the participation of the children in developing hobbies within the hospital, such as the frequency of reading, listening to music, playing games and drawing, and evaluate the implementation effect of this measure. The head nurse randomly selected 20% of the children's records every month and compared them with the actual observation results (obtained through communication with the children and their parents as well as on-site inspections). They checked the accuracy of the records and, in case of any inaccuracies, promptly communicated with the responsible nurses and required them to make corrections.

#### 3.2.2 At home

(A) Gratitude expression and sharing (one by one in WeChat group), the responsible nurse issued a group announcement in the group at 8 am every day, encouraging the children and their parents to do something within their power to their relatives and friends, and report to the group.
  Every day, we assigned dedicated personnel to monitor the interactions within the group chat and used a consistency monitoring record sheet to meticulously record the time when the responsible nurse issued the notification, down to the minute. At the same time, we recorded the reactions of the patients and their parents, such as whether they reported what they did for relatives and friends on time. For those who reported on time, we record the specific content of the report; for those who did not report, we record the situation of non-reporting and the subsequent reminder measures. The head nurse randomly selected 30% of the group chat records every week and conducted a review using a consistency checklist. The review covered whether the notification time was accurate, the completeness and authenticity of the reports made by patients and parents (by conducting private communications with some patients and parents for verification). For any issues identified during the review, timely communication was held with the responsible nurses and rectification was required.
(B) Gratitude diary and visit (WeChat group), the responsible nurse encouraged children and parents to record grateful people or things at 11 noon every day.
  We assigned specific personnel to monitor the WeChat group. We used a consistency monitoring record sheet to record the time when children and parents wrote their gratitude diaries, as well as the richness of the content and the degree of emotional expression. For those who failed to record on time, we would record the situation and promptly send a private message to remind them. The head nurse randomly selected 25% of the gratitude journal entries every two weeks and reviewed them using a consistency checklist. The checklist checked whether the recording time was in accordance with the requirements, whether the content focused on the person or event being grateful for, and whether the emotional expression was genuine. For entries that did not meet the requirements, communication was held with the responsible nurse and they were asked to remind the relevant members to re-record.
(C) Interest training (WeChat group), the responsible nurse encouraged children and parents to record and report daily activities related to interests and hobbies in the group, and sent notes on physical activity for children with asthma in the group from time to time.
  We assigned dedicated personnel to monitor the group chat. We used a consistency monitoring record sheet to record the time and content of the physical activity prompts sent by the responsible nurses, as well as the frequency and detail of the children and parents reporting their interest activities. For those who failed to report on time or whose reports were brief, we would promptly send a private message to remind them and inquire about the reasons. The head nurse randomly selected 20% of the group chat records every month and used a consistency checklist to review them. The checklist checked whether the sports activity notifications were sent on time, whether the content was accurate and appropriate, and whether the reported interests and hobbies activities of the children and parents were true and detailed. For any problems identified, communication was held with the responsible nurse and improvement was requested.
(D) Popular science tweets (WeChat group), 2 times/week, 2 asthma-related tweets were sent regularly within the group. We established a table to record the push notifications of popular science posts, detailing the time, topic, number of views, likes, and comments for each post. By analyzing these data, we could understand the attention and interests of the patients and their parents towards the popular science posts. Based on the feedback results, we adjusted the content and format of the posts. From time to time, small tests related to the science popularization posts were released in the group chat to assess the mastery of asthma knowledge by the children and their parents, and to evaluate the effectiveness of the science popularization posts.
  We assigned specific personnel to record the relevant data of the push notifications for popular science posts, and used data analysis tables to conduct regular analyses of the page views, likes, and comments. At the same time, we also recorded the release time, participation numbers, and correct answer rates of the small-scale tests. The head nurse conducted random checks on the recorded data and analysis results every two weeks, using a consistency checklist for verification. She examined whether the data records were accurate and whether the analysis was reasonable. For small tests, she checked whether the question settings were reasonable and whether the evaluation results were accurate. For any issues identified, she communicated with the responsible personnel and requested a re-analysis or adjustment of the test content.
(E) Regular group interaction (WeChat group), 2 times/week, five responsible nurses took turns on duty and answered questions from 7 pm to 8 pm every Wednesday and Saturday. The on-duty nurses were required to fill in the duty log after each group interaction, including the number of questions answered, the types of questions, the satisfaction of the children and their parents.
  When the on-duty nurse filled out the duty log, they ensured that the recorded information was accurate and complete. They used a unified duty log template to detail the specific circumstances of each issue. The head nurse conducted monthly evaluations of the duty logs, using a consistency checklist. The evaluation covered whether the on-duty nurses accurately and promptly answered the questions as per the protocol (verified through communication with some children and their parents), whether the classification of the questions was reasonable, and whether the records of the children's and parents' satisfaction were true. For any issues identified during the evaluation, discussions were held with the on-duty nurses and they were required to make corrections. Additionally, 10% of the group interaction conversation recordings (if available) were randomly selected for re-listening to further verify the accuracy of the duty logs.
(F) Expert lecture (lecture), once a month, an asthma professional doctor was invited to carry out live online lecture. During the lecture, the participation of the children and their parents was recorded through online sign-in and interactive questioning. After the lecture, their feedback was collected to understand their satisfaction with the lecture content, the expert's presentation style, as well as their expectations and suggestions for future lectures.
  We assigned specific personnel to handle online sign-in and record the interaction questions. We used the lecture participation record form to detail the sign-in time and question content of each participant. After the lecture, we promptly collected the feedback from the participants and used the feedback collection form to record their evaluations of various aspects of the lecture. After each lecture, the head nurse reviewed the recorded and collected materials, using a consistency checklist. She checked whether the attendance records were accurate, whether the interaction question records were complete, and whether the feedback collection was comprehensive. She analyzed the feedback content and assesses whether the lecture effect had reached the expected goal. For any problems identified, she communicated with the lecture organizers and requested improvements.
(G) Directed communication (telephone/WeChat), once a month, the responsible nurse followed up the condition of the children and the implementation of the intervention, encouraged the children and their parents to implement the intervention according to the doctor's advice, gave targeted suggestions for problems in the intervention, and reminded the outpatient review on a regular basis. The responsible nurse promptly recorded the time, content, changes in the child's condition, implementation of intervention measures, problems encountered and proposed solutions after each targeted communication. They established a communication record file, regularly tracked and analyzed the records, evaluated the effectiveness of the targeted communication, promptly identified problems existing in the intervention process and adjust the intervention strategies.
  After the communication was completed, the responsible nurse was required to use a unified communication record template to detail all the contents, ensuring the accuracy of the information. The head nurse reviewed the communication record files monthly using a consistency checklist. The review covered whether the recording time was accurate, whether the content was complete, and whether the description of the child's condition and intervention measures was objective. At the same time, 15% of the communication records were randomly selected and verified for their authenticity through communication with the child and their parents. For any issues identified during the review, discussions were held with the responsible nurses and they were requested to re-record or adjust the intervention strategies.

#### 3.2.3 Language usage

In this study, the main language used for the interviews with patients was Mandarin Chinese. Before the interviews, we informed all the parents of the children participating in the interviews that the interviews would be conducted in Mandarin Chinese and asked if they had any language communication difficulties. For those parents with slight language impairments (such as having a local accent but not affecting basic communication), the interviewers ensured smooth communication by patiently listening and repeating key information. If there were parents who did not understand Mandarin Chinese at all, we would arrange for a translator who was familiar with the local dialect and had some medical knowledge to assist with the interviews. The translators would receive prior training to be familiar with the interview content and procedures to ensure the accuracy and professionalism of the translations.

The communication with healthcare providers (including doctors, nurses) was mainly conducted in Chinese. Within the research team, all members had excellent Chinese communication skills and were able to have clear and accurate exchanges with healthcare providers. Before the communication, the research team prepared relevant research materials and a list of questions to ensure that the communication content was clear and well-organized.

During the communication process, if the healthcare provider used technical terms or had a complex expression, the research team members promptly requested the provider to provide explanations and clarifications to ensure a correct understanding of the information. At the same time, the research team also introduced the purpose, methods, and progress of the research to the healthcare provider in simple and understandable language, promoting effective communication and cooperation between the two parties. For possible language differences in habits, both parties coordinated through a respectful and inclusive attitude to avoid any impact on the smooth progress of the research due to language issues.

Implementation methods of peer interactive education: Children with asthma in the group were gathered for collective or group peer interactive education every day, including knowledge dissemination based on heuristic questioning and mutual assistance, self-experience sharing of rhinitis and asthma symptoms, mutual teaching of operation methods of inhalation devices and peak current meter, symptom self-monitoring and mutual monitoring, and mutual diagnosis and treatment of peers with symptoms. During the study period, the treatment of asthma symptoms and the use of various asthma drug inhalation devices were explained by examples when the companions had acute asthma attacks and allergic symptoms. The children demonstrated how to use the peak current meter, and the peer helped to correct the error. Children were not supervised by parents throughout the whole process, and peer partners supported each other, encouraged each other, and helped each other. After discharge, the children carried out peer interactive education once every month.

The duration of both interventions were 6 months.

## 4 Observation indicators

1. Self-management ability: The Chinese version of asthma self-management scale for children aged 7–17 years was used before intervention, 6 months and 12 months after intervention, respectively (16). The scale included 34 items in 3 dimensions: daily life management, disease medical management and social psychological management. The score range was 34∼170 points, the higher the score, the better the self-management ability.
2. Asthma control score: The Chinese version of Childhood Asthma Control Test (C-ACT) was used to evaluate the asthma control of children in the two groups before intervention, 6 months and 12 months after intervention respectively (17). There were 7 items in the scale, the first 4 items scored 0–3 points, and the last 3 items scored 0–5 points, with a full score of 27 points. The higher the score, the better the asthma control.
3. Lung function: The levels of forced expiratory volume (FEV1), forced vital capacity (FVC) and peak expiratory flow (PEF) in the first second were measured by a pulmonary function detector before intervention, 6 months and 12 months after intervention respectively.
4. Quality of life: The pediatric asthma quality of life questionnaire (PAQLQ) was used before intervention, 6 months and 12 months after intervention, respectively (18). The questionnaire included 23 items, which were divided into 3 dimensions, namely, the activity dimension (5 items), the symptom dimension (10 items) and the emotion dimension (8 items). The total score ranged from 23 to 161, and the higher the score, the higher the quality of life.
5. Recurrence: The frequency of asthma attacks, emergency department and hospitalization were compared between the two groups within 12 months after treatment.
6. Nursing compliance: In the context of this study, “nursing compliance” refers to the degree to which children with asthma follow the established nursing plan and medical instructions, covering multiple key nursing aspects such as medication use, inhaler usage, regular check-ups and reflection, avoidance of trigger factors, and timely follow-up visits. Specifically, medication compliance requires children to accurately take the prescribed dosage, frequency, and time of medication as directed by the doctor; inhaler usage compliance emphasizes that children correctly master the usage method of the inhaler and use it appropriately when necessary; regular check-up and reflection compliance means that children visit the hospital as scheduled at the appointed time for check-ups and actively cooperate with the doctor to reflect on the condition and treatment effect; avoiding trigger factors compliance requires the children and their families to understand and try to avoid factors that may trigger asthma attacks, such as allergens, cold air, and intense exercise; timely follow-up visit compliance means that children visit the hospital for follow-up visits when there are changes in their condition or when it is time for a follow-up visit.
  A self-designed Chinese questionnaire on nursing compliance of asthmatic children was used to evaluate the nursing compliance of children in the two groups 12 months after intervention, including medication, use of inhalers, regular review and reflection, avoidance of triggering factors, and timely follow-up visits, with a full score of 100. The detailed scoring criteria were as follows: (1) Medication administration: 20 points for strictly following the doctor's instructions; 15 points for occasionally missing or taking the wrong medication but without affecting the overall treatment outcome; 10 points for frequently missing or taking the wrong medication and having a certain impact on the treatment outcome; 0 points for almost never following the doctor's instructions. (2) Using inhaler: 20 points for proficiently mastering and correctly using the inhaler; 15 points for being able to use the inhaler correctly under the guidance of medical staff; 10 points for using the inhaler method with many errors and still being unable to correct them even after multiple instructions; 0 points for almost never using the inhaler. (3) Regular follow-ups and reflection: If the children strictly adheres to the scheduled time for follow-ups and actively participates in reflecting on and discussing the condition with the doctor, they will receive 20 points; if they occasionally miss the follow-up time but promptly make up for it and participate in the reflection and discussion, they will receive 15 points; if they frequently miss the follow-up time and is not proactive in reflecting on and discussing the condition, they will receive 10 points; if they hardly ever undergo follow-ups and reflection, they will receive 0 points. (4) Avoidance of triggers: 20 points are awarded if the children can clearly understand and actively avoid all possible asthma triggers; 15 points are given if the children understand some of the triggers and can try to avoid them; 10 points are awarded if the children have limited knowledge of triggers and the avoidance measures are inadequate; 0 points are given if the children have little or no knowledge of triggers and do not take any avoidance measures. (5) Regular follow-up visits: 20 points will be awarded if the children can attend the follow-up visit in a timely manner when there are changes in their condition or when the scheduled time for the visit arrives; 15 points will be awarded if the children occasionally delay the visit due to special circumstances but communicate with the doctor in time; 10 points will be awarded if the children frequently delay the visit without communicating with the doctor; 0 points will be awarded if the children almost never attend the follow-up visit on time.
  Based on the questionnaire scores, the nursing compliance was classified into three levels: <60 was classified as non-compliance, 61–85 as partial compliance, and 86–100 as complete compliance. Total compliance rate = (number of complete compliance cases + number of partial compliance cases)/Total number of cases × 100%.
  During the questionnaire design process, we added some questions related to factors that might affect the compliance of nursing care, including the age and gender of the child, the family's economic status, the parents' educational level, the parents' understanding of asthma disease, and the family support system. Non-compliance may have various impacts on the results of this study. Firstly, when evaluating the effects of intervention measures on indicators such as self-management ability, lung function, and quality of life of children with asthma, non-compliant children may not fully accept the intervention measures, resulting in a lesser improvement in various indicators of non-compliant children within the intervention group compared to compliant children, thereby underestimating the actual effectiveness of the intervention measures. Secondly, non-compliance may increase the variability of the research results, making it difficult to accurately reflect the differences between the two groups and affecting the reliability and stability of the research conclusions.
7. Nursing satisfaction: The Chinese version of Newcastle Nursing Satisfaction Scale was used for assessment. There were 19 items in the scale, and each item was scored from 1 to 5 points. The total score was 95 points, <67 points indicated dissatisfied, 67–85 points indicated satisfied, and >85 points indicated very satisfied. Total satisfaction = (number of very satisfied cases + number of satisfied cases)/Total number of cases × 100%.

## 5 Statistical analysis

SPSS 25.0 software was used for statistical analysis. The statistical data were expressed as rate and *χ*^2^ test was used for comparison between the two groups. Measurement data were expressed in (*x* ± *s*) and two-way repeated-measures ANOVA followed by Bonferroni-Dunn tests was adopted for comparison between the two groups. Effect sizes (Cohen's *d*) and 95% confidence intervals (CI) were calculated. *P* < 0.05 meant the difference was significant.

## 6 Results

### 6.1 General data in both groups

There was no significant difference in general data between the 2 groups (*P* > 0.05, Table 1).

**Table 1:** General data in both groups.

Groups	Cases	Gender		Age (years)	Course of disease (years)
		Male	Female		
Control group	60	30 (50.00)	30 (50.00)	9.23 ± 1.27	1.72 ± 0.18
Study group	60	32 (53.33)	28 (46.67)	9.32 ± 1.32	1.74 ± 0.21
*χ*^2^/*t*		0.13		0.38	0.56
*P*		0.71		0.70	0.57

### 6.2 Self-management ability in both groups

A repeated measures analysis of variance was employed, with “group” (study group, control group) as the between-group factor and “time” (before intervention, 6 months after intervention, 12 months after intervention) as the within-group factor, to analyze the scores of daily life management, disease medical management and social psychological management of the two groups of patients.

For the scores of daily life management, the time main effect was significant [F (1, 354) = 64.18, *P* < 0.05], the group main effect was significant [F (2, 354) = 271.0, *P* < 0.05], and the “group ×  time” interaction effect was significant [F (2, 354) = 17.78, *P* < 0.05].

In the control group, the scores of daily life management after 6 months of intervention was higher than before the intervention (*P* < 0.05), with a Cohen's d value of 1.27; the scores of daily life management after 12 months of intervention was higher than before the intervention (*P* < 0.05), with a Cohen's d value of 2.35.

In the study group, the scores of daily life management after 6 months of intervention was higher than before the intervention (*P* < 0.05), with a Cohen's d value of 2.32; the scores of daily life management after 12 months of intervention was higher than before the intervention (*P* < 0.05), with a Cohen's d value of 3.61.

**Figure 1:**
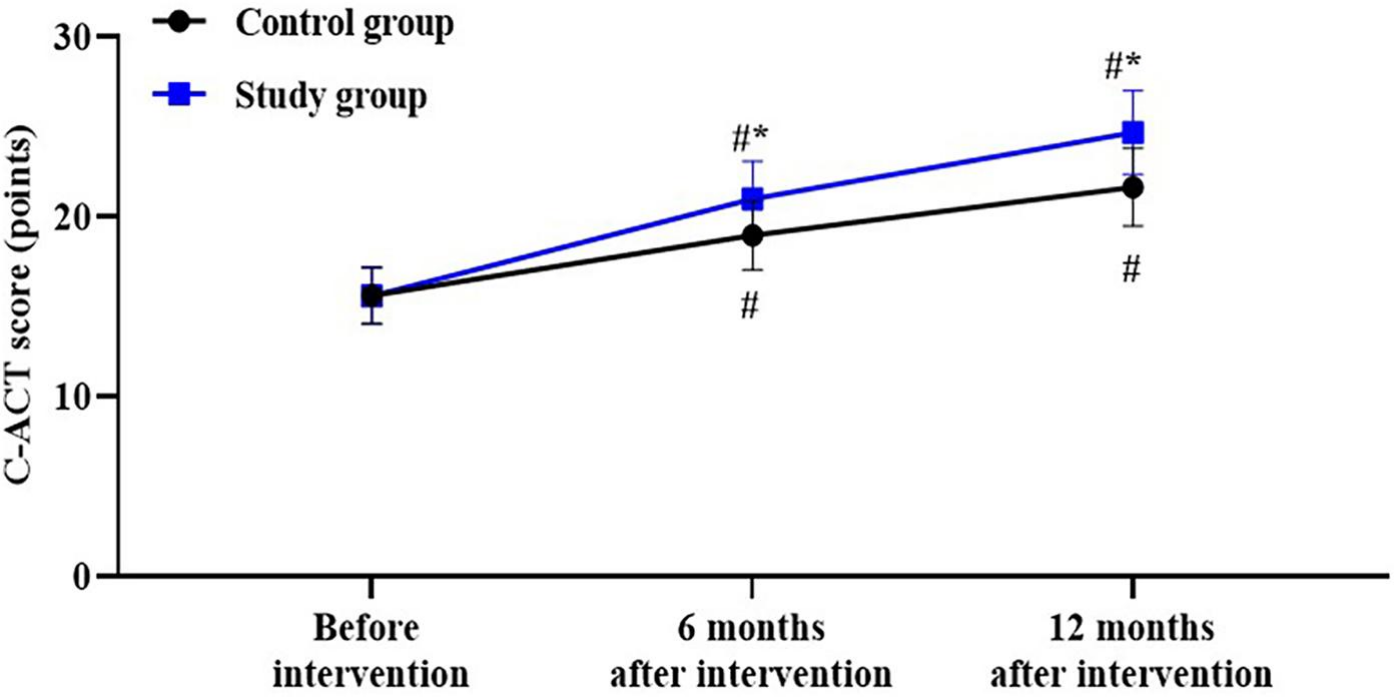
Self-management ability in both groups. ^#^*P* < 0.05, vs. before intervention; *P < 0.05, vs. control group.

Compared to the control group, the study group had higher scores of daily life management at 6 and 12 months after intervention (*P* < 0.05), with a Cohen's d value of 1.05 and 1.30, respectively (Figure 1).

For the scores of disease medical management, the time main effect was significant [F (1, 354) = 25.61, *P* < 0.05], the group main effect was significant [F (2, 354) = 109.1, *P* < 0.05], and the “group × time” interaction effect was significant [F (2, 354) = 6.68, *P* < 0.05].

In the control group, the scores of disease medical management after 6 months of intervention was higher than before the intervention, with a Cohen's d value of 0.60; the scores of disease medical management after 12 months of intervention was higher than before the intervention, with a Cohen's d value of 1.51.

In the study group, the scores of disease medical management after 6 months of intervention was higher than before the intervention, with a Cohen's d value of 1.36; the scores of disease medical management after 12 months of intervention was higher than before the intervention, with a Cohen's d value of 2.28.

Compared to the control group, the study group had higher scores of disease medical management at 6 and 12 months after intervention (*P* < 0.05), with a Cohen's d value of 0.75 and 0.78, respectively (Figure 1).

For the scores of social psychological management, the time main effect was significant [F (1, 354) = 49.94, *P* < 0.05], the group main effect was significant [F (2, 354) = 257.0, *P* < 0.05], and the “group × time” interaction effect was significant [F (2, 354) = 13.11, *P* < 0.05].

In the control group, the scores of social psychological management after 6 months of intervention was higher than before the intervention, with a Cohen's d value of 1.24; the scores of social psychological management after 12 months of intervention was higher than before the intervention, with a Cohen's d value of 2.40.

In the study group, the scores of social psychological management after 6 months of intervention was higher than before the intervention, with a Cohen's d value of 2.26; the scores of social psychological management after 12 months of intervention was higher than before the intervention, with a Cohen's d value of 3.41.

Compared to the control group, the study group had higher scores of social psychological management at 6 and 12 months after intervention (*P* < 0.05), with a Cohen's d value of 1.03 and 1.06, respectively (Figure 1).

### 6.3 Asthma control score in both groups

A repeated measures analysis of variance was employed, with “group” (study group, control group) as the between-group factor and “time” (before intervention, 6 months after intervention, 12 months after intervention) as the within-group factor, to analyze the C-ACT score of the two groups of patients.

The time main effect was significant [F (1, 354) = 65.36, *P* < 0.05], the group main effect was significant [F (2, 354) = 450.6, *P* < 0.05], and the “group × time” interaction effect was significant [F (2, 354) = 19.12, *P* < 0.05].

In the control group, the C-ACT score after 6 months of intervention was higher than before the intervention (*P* < 0.05), with a Cohen's d value of 1.90; the C-ACT score after 12 months of intervention was higher than before the intervention (*P* < 0.05), with a Cohen's d value of 3.20.

In the study group, the C-ACT score after 6 months of intervention was higher than before the intervention (*P* < 0.05), with a Cohen's d value of 2.91; the C-ACT score after 12 months of intervention was higher than before the intervention (*P* < 0.05), with a Cohen's d value of 4.59.

**Figure 2:**
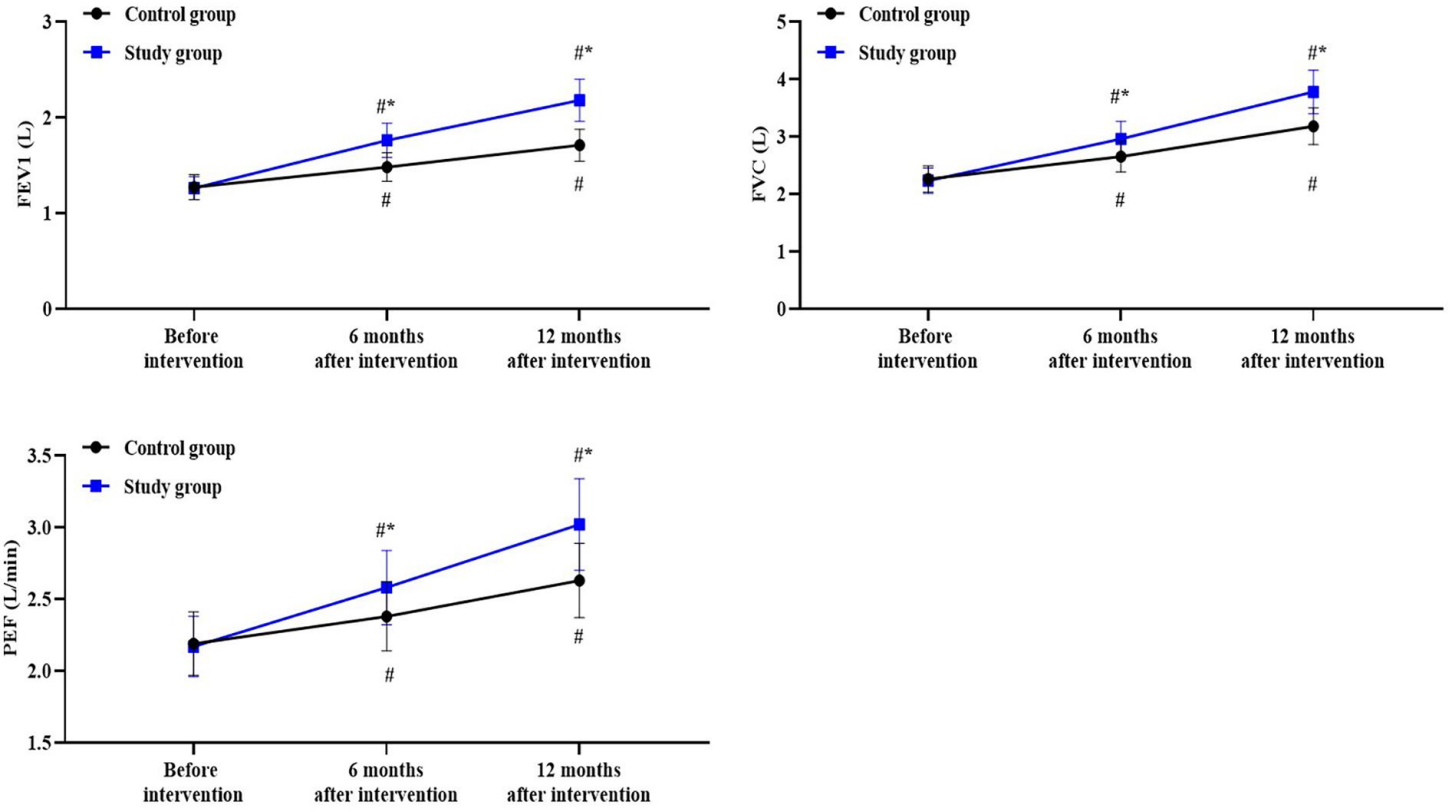
Asthma control score in both groups. ^#^*P* < 0.05, vs. before intervention; *P < 0.05, vs. control group.

Compared to the control group, the study group had higher the C-ACT score at 6 and 12 months after intervention (*P* < 0.05), with a Cohen's d value of 1.00 and 1.35, respectively (Figure 2).

### 6.4 Lung function in both groups

A repeated measures analysis of variance was employed, with “group” (study group, control group) as the between-group factor and “time” (before intervention, 6 months after intervention, 12 months after intervention) as the within-group factor, to analyze the levels of FEV1, FVC and PEF of the two groups of patients.

For the levels of FEV1, the time main effect was significant [F (1, 354) = 201.0, *P* < 0.05], the group main effect was significant [F (2, 354) = 509.4, *P* < 0.05], and the “group × time” interaction effect was significant [F (2, 354) = 64.33, *P* < 0.05].

In the control group, the levels of FEV1 after 6 months of intervention was higher than before the intervention (*P* < 0.05), with a Cohen's d value of 1.49; the levels of FEV1 after 12 months of intervention was higher than before the intervention (*P* < 0.05), with a Cohen's d value of 2.90.

In the study group, the levels of FEV1 after 6 months of intervention was higher than before the intervention (*P* < 0.05), with a Cohen's d value of 3.26; the levels of FEV1 after 12 months of intervention was higher than before the intervention (*P* < 0.05), with a Cohen's d value of 5.19.

**Figure 3:**
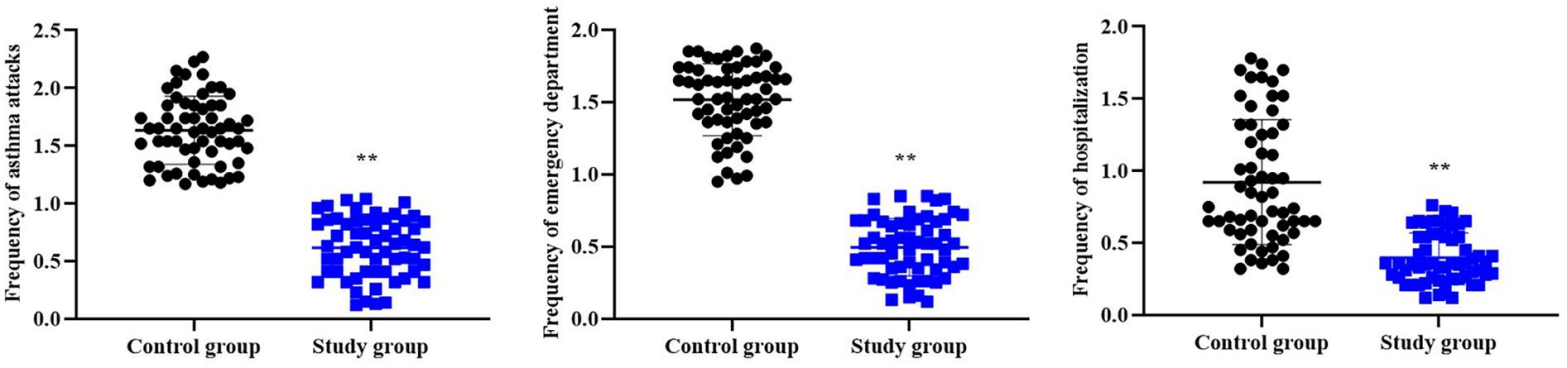
Lung function in both groups. ^#^*P* < 0.05, vs. before intervention; *P < 0.05, vs. control group.

Compared to the control group, the study group had higher levels of FEV1 at 6 and 12 months after intervention (*P* < 0.05), with a Cohen's d value of 1.69 and 2.39, respectively (Figure 3).

For the levels of FVC, the time main effect was significant [F (1, 354) = 89.85, *P* < 0.05], the group main effect was significant [F (2, 354) = 532.5, *P* < 0.05], and the “group × time” interaction effect was significant [F (2, 354) = 34.61, *P* < 0.05].

In the control group, the levels of FVC after 6 months of intervention was higher than before the intervention (*P* < 0.05), with a Cohen's d value of 1.55; the levels of FVC after 12 months of intervention was higher than before the intervention (*P* < 0.05), with a Cohen's d value of 3.30.

In the study group, the levels of FVC after 6 months of intervention was higher than before the intervention (*P* < 0.05), with a Cohen's d value of 2.71; the levels of FVC after 12 months of intervention was higher than before the intervention (*P* < 0.05), with a Cohen's d value of 4.99.

Compared to the control group, the study group had higher levels of FVC at 6 and 12 months after intervention (*P* < 0.05), with a Cohen's d value of 1.06 and 1.70, respectively (Figure 3).

For the levels of PEF, the time main effect was significant [F (1, 354) = 50.28, *P* < 0.05], the group main effect was significant [F (2, 354) = 193.5, *P* < 0.05], and the “group × time” interaction effect was significant [F (2, 354) = 19.55, *P* < 0.05].

In the control group, the levels of PEF after 6 months of intervention was higher than before the intervention (*P* < 0.05), with a Cohen's d value of 0.82; the levels of PEF after 12 months of intervention was higher than before the intervention (*P* < 0.05), with a Cohen's d value of 1.82.

In the study group, the levels of PEF after 6 months of intervention was higher than before the intervention (*P* < 0.05), with a Cohen's d value of 1.73; the levels of PEF after 12 months of intervention was higher than before the intervention (*P* < 0.05), with a Cohen's d value of 3.14.

Compared to the control group, the study group had higher levels of PEF at 6 and 12 months after intervention (*P* < 0.05), with a Cohen's d value of 0.79 and 1.33, respectively (Figure 3).

### 6.5 Quality of life in both groups

A repeated measures analysis of variance was employed, with “group” (study group, control group) as the between-group factor and “time” (before intervention, 6 months after intervention, 12 months after intervention) as the within-group factor, to analyze the scores of activity dimension, symptom dimension and emotional dimension of the two groups of patients.

For the scores of activity dimension, the time main effect was significant [F (1, 354) = 182.8, *P* < 0.05], the group main effect was significant [F (2, 354) = 335.7, *P* < 0.05], and the “group × time” interaction effect was significant [F (2, 354) = 50.72, *P* < 0.05].

In the control group, the scores of activity dimension after 6 months of intervention was higher than before the intervention (*P* < 0.05), with a Cohen's d value of 1.21; the scores of activity dimension after 12 months of intervention was higher than before the intervention (*P* < 0.05), with a Cohen's d value of 2.22.

In the study group, the scores of activity dimension after 6 months of intervention was higher than before the intervention (*P* < 0.05), with a Cohen's d value of 2.94; the scores of activity dimension after 12 months of intervention was higher than before the intervention (*P* < 0.05), with a Cohen's d value of 4.33.

**Figure 4:**
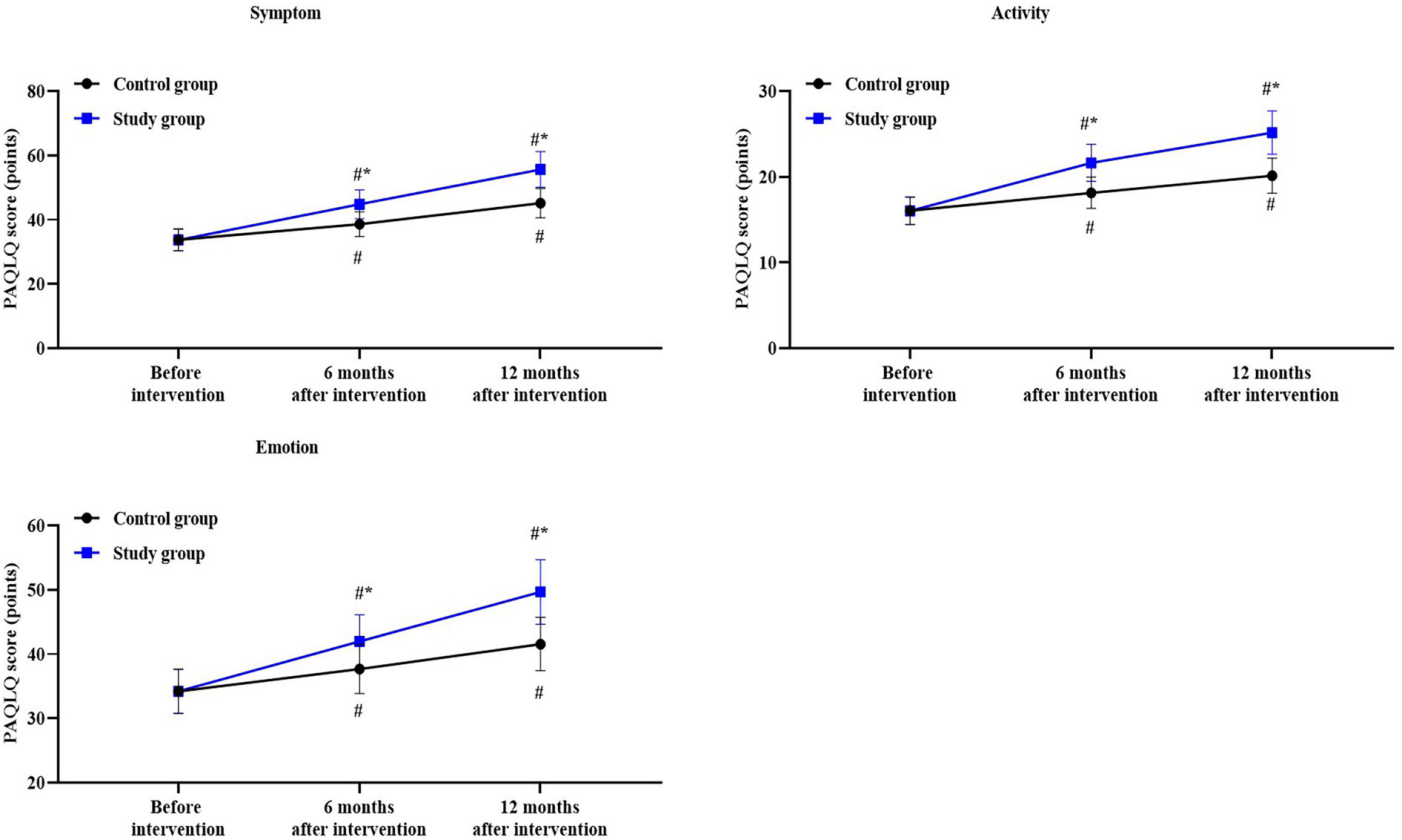
Quality of life in both groups. ^#^*P* < 0.05, vs. before intervention; *P < 0.05, vs. control group.

Compared to the control group, the study group had higher scores of activity dimension at 6 and 12 months after intervention (*P* < 0.05), with a Cohen's d value of 1.74 and 2.18, respectively (Figure 4).

For the scores of symptom dimension, the time main effect was significant [F (1, 354) = 152.5, *P* < 0.05], the group main effect was significant [F (2, 354) = 458.9, *P* < 0.05], and the “group × time” interaction effect was significant [F (2, 354) = 46.08, *P* < 0.05].

In the control group, the scores of symptom dimension after 6 months of intervention was higher than before the intervention (*P* < 0.05), with a Cohen's d value of 1.35; the scores of symptom dimension after 12 months of intervention was higher than before the intervention (*P* < 0.05), with a Cohen's d value of 2.86.

In the study group, the scores of symptom dimension after 6 months of intervention was higher than before the intervention (*P* < 0.05), with a Cohen's d value of 2.81; the scores of symptom dimension after 12 months of intervention was higher than before the intervention (*P* < 0.05), with a Cohen's d value of 4.77.

Compared to the control group, the study group had higher scores of symptom dimension at 6 and 12 months after intervention (*P* < 0.05), with a Cohen's d value of 1.48 and 2.06, respectively (Figure 4).

For the scores of emotion dimension, the time main effect was significant [F (1, 354) = 94.47, *P* < 0.05], the group main effect was significant [F (2, 354) = 240.3, *P* < 0.05], and the “group × time” interaction effect was significant [F (2, 354) = 30.67, *P* < 0.05].

In the control group, the scores of emotion dimension after 6 months of intervention was higher than before the intervention (*P* < 0.05), with a Cohen's d value of 0.95; the scores of emotion dimension after 12 months of intervention was higher than before the intervention (*P* < 0.05), with a Cohen's d value of 1.92.

In the study group, the scores of emotion dimension after 6 months of intervention was higher than before the intervention (*P* < 0.05), with a Cohen's d value of 2.04; the scores of emotion dimension after 12 months of intervention was higher than before the intervention (*P* < 0.05), with a Cohen's d value of 3.60.

Compared to the control group, the study group had higher scores of emotion dimension at 6 and 12 months after intervention (*P* < 0.05), with a Cohen's d value of 1.08 and 1.75, respectively (Figure 4).

### 6.6 Recurrence in both groups

**Figure 5:**
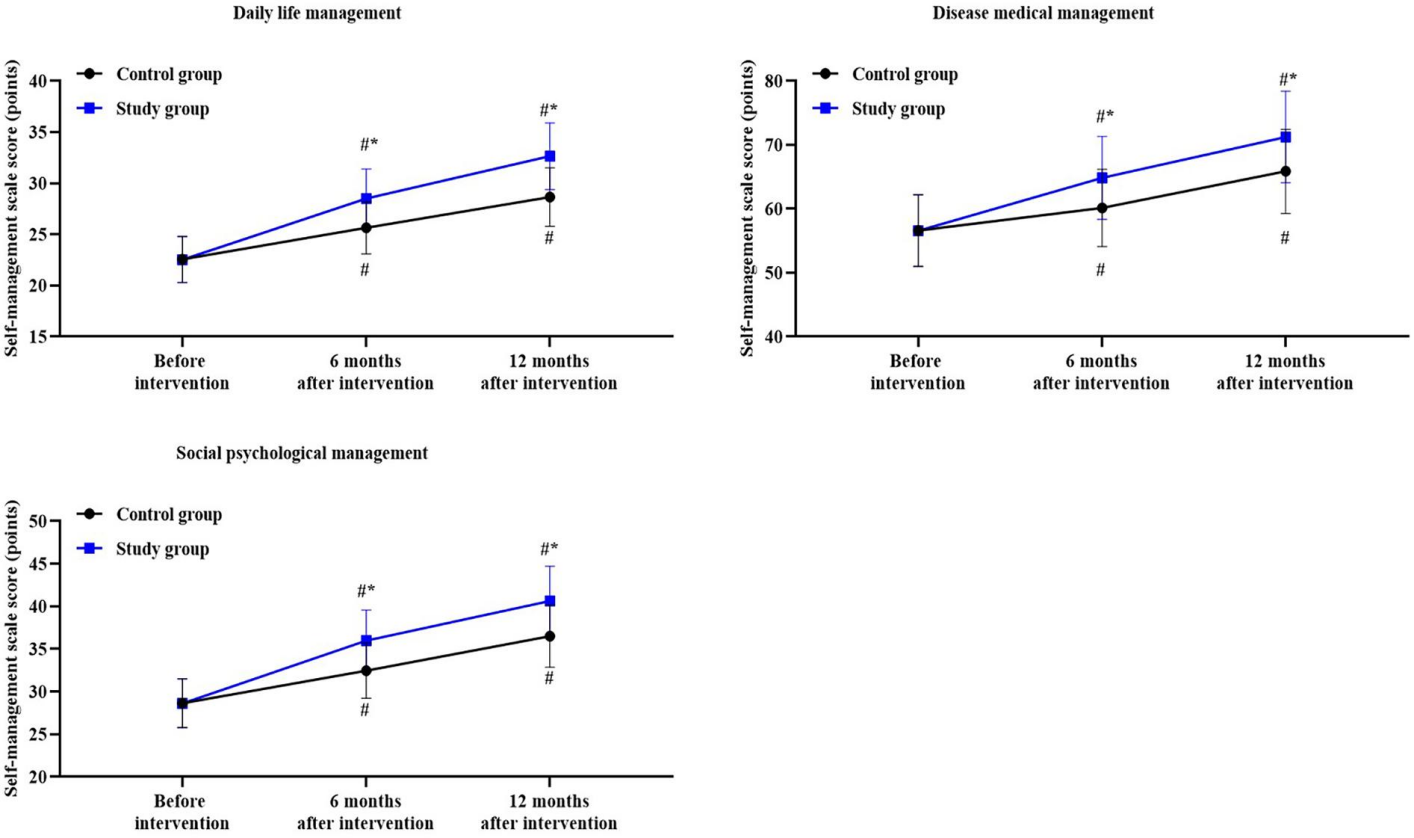
Recurrence in both groups. **P < 0.01.

Compared to the control group, the study group had lower frequency of asthma attacks, emergency department and hospitalization within 12 months after treatment (*P* < 0.01), with a Cohen's d value of 3.77, 4.68 and 1.98, respectively (Figure 5).

### 6.7 Nursing compliance in both groups

The total nursing compliance of the control group was 83.34% (50/60), and that of the study group was 96.66% (58/60). Compared to the control group, the study group had better nursing compliance (*P* < 0.05, Table 2).

**Table 2:** Nursing compliance in both groups.

Groups	Cases	Complete compliance	Partial compliance	Non-compliance	Total compliance rate
Control group	60	28 (46.67)	22 (36.67)	10 (16.66)	50 (83.34)
Study group	60	35 (58.33)	23 (38.33)	2 (3.34)	58 (96.66)
*χ*^2^/*t*					5.92
*P*					0.01

### 6.8 Nursing satisfaction in both groups

The total satisfaction rate of the control group was 81.66% (49/60), and that of the study group was 96.66% (58/60). Compared to the control group, the study group had better nursing satisfaction (*P* < 0.05, Table 3).

**Table 3:** Nursing satisfaction in both groups.

Groups	Cases	Very satisfied	Satisfied	Dissatisfied	Total satisfaction rate
Control group	60	26 (43.33)	23 (38.33)	11 (18.34)	49 (81.66)
Study group	60	32 (53.33)	26 (42.33)	2 (3.34)	58 (96.66)
*χ*^2^/*t*					6.98
*P*					0.01

## 7 Discussion

The gratitude extension-construction theory emphasizes perceived benefits and bestowed benefits, where perceived benefits refer to the benefits individuals receive from others, and bestowed benefits refer to the benefits individuals bring by giving back and serving others (19). Together, these two aspects form the basis of gratitude and can help individuals better understand and experience the positive effects of gratitude (20).

In our study, the results indicated that compared with the control group, the study group had higher scores of daily life management, disease medical management and social psychological management at 6 and 12 months after intervention. These results implied that the combination of the gratitude extension-construction theory and peer interactive education could promote the self-management ability of school-age children with asthma. The characteristics of peer interactive asthma education are that children are completely separated from their parents' supervision, live in a strange foreign environment, and get along with their peers day and night to carry out collective propaganda and interactive practice of asthma knowledge, and use the examples of peer asthma attacks for practical education, so as to promote children to give full play to their subjective initiative for learning and self-management under the condition that they cannot rely on their parents (21). Besides, gratitude extension-construction theory can make children have a higher cognition of the disease and improve self-management ability (22).

Besides, our study indicated that compared with the control group, the study group had higher C-ACT score, higher levels of FEV1, FVC and PEF, higher scores of activity dimension, symptom dimension and emotional dimension at 6 and 12 months after intervention. Meanwhile, compared with the control group, the study group had a lower frequency of asthma attacks within 12 months after treatment, fewer emergency visits and hospitalizations, better nursing compliance, and higher nursing satisfaction. These results implied that the combination of the gratitude extension-construction theory and peer interactive education could better control asthma, improve lung function, promote the quality of life, reduce the recurrence of asthma, and enhance the nursing compliance and nursing satisfaction of school-age children with asthma. The gratitude extension-construction theory considers both in-hospital nursing and post-discharge nursing for children. During hospitalization, motivational lectures are organized to enhance children's understanding of asthma-related knowledge, make them realize the importance of medication as prescribed by doctors, improve their medication compliance, and promote the lung function and quality of life (23). In addition, children can learn and exchange experience and experience of asthma disease with their peers, learn asthma knowledge from medical professionals, and express and discuss their understanding of asthma with each other.

Our research has some limitations. First, the number of samples included in this study is relatively limited, which may lead to deviations between the data results and the actual values. The small sample size is insufficient to fully represent the diverse characteristics of the large group of school-age children with asthma. In statistical analysis, a small sample size can also lead to insufficient statistical power, making it difficult to detect some actual differences that do exist but have relatively small effects. This undermines the robustness of the research conclusions. Second, this study is a single-center study. The selected samples are solely from a specific medical institution or region and do not have broad representativeness. Factors such as the distribution of medical resources, disease management models, and cultural backgrounds in different regions may all have an impact on the condition and treatment outcome of school-age children with asthma. Furthermore, single-center studies may be influenced by the specific medical procedures and care standards of that institution, which further limits the representativeness of the sample. Third, in terms of evaluating nursing compliance, the questionnaire used was self-designed. Although efforts were made to refer to relevant literature and expert opinions during the design process, this questionnaire has not yet undergone large-scale and multi-center pre-validation. Due to the lack of extensive sample testing and expert validation, this questionnaire may not comprehensively and accurately cover all the key elements of nursing compliance. Moreover, the reliability and validity of the questionnaire have not been fully verified, and there may be measurement errors, thereby affecting the comprehensiveness and accuracy of the assessment of nursing compliance. In addition, this study adopted a partial blinding strategy in its design. Specifically, when recruiting children and their parents, the specific information about the group allocation was not disclosed. Only that they would participate in an educational intervention study for children with asthma was informed. At the same time, it was ensured that the parents were unaware of the specific group allocation of the children throughout the study period. The result evaluators were not involved in the group allocation and intervention implementation process, and the identifiers that might reveal the group information were removed before the evaluation. Although this design largely reduced the influence of expected biases and subjective attitudes on the research results, and improved the objectivity and fairness of the evaluation, we must admit that achieving complete “double blinding” in behavioral intervention studies is extremely challenging. Specifically, although the participants and the caregivers (parents) were not informed of the details of the groups, children might indirectly perceive their group assignment during the process of receiving different educational methods. This is because of differences in the educational content and the way they interact with the educators. Additionally, although parents were not informed of the relationship between the information and the group assignment when providing routine care and participating in information collection, their observations and feedback on their children's daily behaviors might still be influenced by potential subjective factors, although this influence was designed to be minimized. Therefore, although this study took several measures to minimize performance deviations to the greatest extent possible, it is impossible to completely rule out the possibility of their existence.

Future research should expand the sample size and conduct multi-center studies. By including more school-age children with asthma from different regions and different medical institutions, the representativeness of the sample and the generalizability of the research results can be improved. Multi-center studies can reduce the influence of specific factors from a single institution on the research results, making the results more reflective of the real situations in different cultures and medical environments. In addition, a large sample size can also increase the statistical power, enabling the research to detect more subtle differences and provide more reliable evidence for clinical practice. In view of the limitations of the nursing compliance assessment questionnaire, future research should develop standardized assessment tools that have been pre-validated on a large scale and in multiple centers. During the design of the questionnaire, all key dimensions of nursing compliance should be fully considered, and experts in the relevant field should be invited for argumentation and revision. At the same time, extensive sample tests should be conducted to evaluate the reliability and validity of the questionnaire, ensuring that it can accurately and comprehensively assess the nursing compliance of children. In addition, it is also possible to explore the use of a combination of multiple assessment methods, such as combining electronic monitoring equipment to record the medication situation of children, to improve the accuracy and objectivity of the assessment.

In future research, measures for data quality control should be strengthened. During the data collection process, researchers are uniformly trained to ensure they master the correct data collection methods and standards. At the same time, a strict data review mechanism is established to regularly inspect and verify the collected data, promptly identifying and correcting errors and omissions in the data. Additionally, a two-person data entry method can be adopted to enhance the accuracy of the data. To comprehensively evaluate the long-term effects of the intervention measures combining the extended gratitude construction theory with peer interaction education, future research should conduct long-term follow-ups. By extending the follow-up period, the long-term changes in indicators such as children's self-management ability, lung function, quality of life, recurrence rate, nursing compliance, and nursing satisfaction can be observed, providing more comprehensive evidence for clinical practice. At the same time, long-term follow-ups can also identify any potential long-term adverse reactions or problems of the intervention measures, providing a basis for further optimizing the intervention plan. The management of school-age children with asthma involves multiple disciplines, such as pediatrics, nursing, psychology, and sociology. Future research should strengthen interdisciplinary collaboration, fully leveraging the strengths of each discipline, and jointly design and implement research plans. Through interdisciplinary cooperation, the comprehensiveness and scientific nature of the research can be enhanced, providing more comprehensive and effective care and management plans for school-age children with asthma.

Future research can further explore more refined blind design, or combine other research methods (such as objective physiological indicator monitoring) to more comprehensively evaluate the intervention effect, in order to provide more scientific and reliable basis for educational intervention for children with asthma.

Moreover, we plan to establish a multidisciplinary research team consisting of pediatric experts, pharmacists, and public health scholars, to ensure the comprehensiveness and professionalism of the review. The research team will systematically collect and analyze the guidelines issued by authoritative medical institutions in the United States, the results of large-scale clinical trials, and actual clinical case data. At the same time, we will also pay attention to the innovative models in the management of childhood asthma in the United States, such as remote monitoring and intervention based on mobile medical technology, and the comprehensive management model with multidisciplinary collaboration, in order to provide new ideas and methods for our research.

In our subsequent research, we plan to delve deeper into the relationship between the social determinants of health and the self-management ability and quality of life of school-age children with asthma. Through qualitative research methods, such as conducting in-depth interviews and home visits with the families of children who frequently visit the emergency room, we aim to understand the actual difficulties and needs they encounter in disease management, and explore the underlying reasons that influence the self-management behaviors of the children. At the same time, we will also combine quantitative research methods to analyze the correlations between different social determinants and the self-management ability and quality of life indicators of the children, in order to provide scientific basis for formulating targeted intervention strategies.

## 8 Conclusion

Our study demonstrates that the combination of the gratitude extension-construction theory and peer interactive education significantly promote the self-management ability, improve lung function, promote the quality of life, reduce the recurrence, and enhance the nursing compliance and nursing satisfaction of school-age children with asthma in China.

## Data Availability

The datasets presented in this study can be found in online repositories. The names of the repository/repositories and accession number(s) can be found in the article/Supplementary Material.
